# Editorial: Microbial chain elongation—carbon recovering biorefineries for the circular economy

**DOI:** 10.3389/fbioe.2024.1448975

**Published:** 2024-07-24

**Authors:** David P. B. T. B. Strik, Ramon Ganigue

**Affiliations:** ^1^ Environmental Technology, Wageningen University and Research, Wageningen, Netherlands; ^2^ UNLOCK, Wageningen University and Research, Wageningen, Netherlands; ^3^ Center for Microbial Ecology and Technology (CMET), Faculty of Bioscience Engineering, Ghent University, Ghent, Belgium; ^4^ Centre for Advanced Process Technology for Urban Resource Recovery (CAPTURE), Ghent, Belgium

**Keywords:** chain elongation, biorefinery, resource recovery, anaerobic digestion, bioprocess engineering, circular economy, microbiology

In the quest for sustainability, the notion of closing the carbon loop in our society has emerged as a paramount objective. Once seen as a burden, waste, residues, “used products,” and CO_2_ are now recognized as crucial inputs to foster the Circular Economy. This shift in perspective underscores the necessity to develop novel technologies to convert these streams into new commodity chemicals or circular products, thereby transforming challenges into opportunities.

Microbial Chain Elongation stands at the forefront of this endeavor, offering a promising pathway to advance the utilization of waste streams and CO_2_ for the production of valuable short and medium chain carboxylate acids (i.e., SCCA and MCCA). The chain elongation processes are part of the so-called Carboxylate platform which enables the production of carboxylates and other biochemicals (Spirito et al., 2014; Angenent et al., 2016; Holtzapple et al., 2022). Chemicals from this platform can be used as such or can be further processed to serve many different applications ranging from niche specialty use in pharmaceuticals to bulk use in the manufacturing of biodegradable plastics or e-fuels. Many entrepreneurs are leading the way in commercialization by establishing commercial-scale factories with production capacities in the order of several tons per year (Wageningen University and Research, 2021; Afyren, 2024). Still, the fundamentals of this endeavor and the process development, such as the use of new feedstocks, are of research interest. The seven research articles presented in this Research Topic encapsulate the multifaceted aims and objectives that are driving the advancement of this innovative field.

Understanding Microbial Ecology: Establishing microbial dynamics and interactions in chain elongation systems is pivotal for optimizing production processes. Through microbial community analysis and functional profile prediction, researchers delve into the intricacies of bacterial communities involved in chain elongation from various substrates such as food waste extract and (as later on presented in the editorial), ultra-filtered milk permeate. The microbiome analysis revealed certain key players for both fermentative and chain-elongating key players. These findings not only improve our understanding of microbiome functionality but also pave the way for tailored engineering of high-performance microbial consortia (Crognale et al.).

Metagenomic Insights: Leveraging metagenome-level analyses, researchers unravel the genomic features underpinning the synthesis of target products within fermentative microbial communities from a 282-day bioreactor experiment. By identifying key microbial players and their metabolic pathways, this study provided invaluable guidance for the design and operation of bioprocesses aimed at converting low-value coproducts like fed ultra-filtered milk permeate into carboxylic acids (Walters et al.).

Common Models of Community Organization: Comparative genomic analyses shed light on the common organizational principles governing microbial communities in different agro-industrial residues. The research revealed that common biological functions among the microbial communities in different bioreactors led to different enrichment of microbial communities depending on the agro-industrial residue tested. Nevertheless, the results supported the conclusion that the microbial ecology model tested was suitable to explain the MCCA production potential from all residues studied. Understanding the core-functional groups of carboxylic acids and chain elongation microbiomes continues to be an important field (Myers et al.).

Optimizing Growth Strategies: The growth characteristics of key functional guilds in sugar-based chain elongation systems, such as lactic acid bacteria and chain-elongating microbes, show that they follow differential ecological strategies. Understanding these features and how they drive community dynamics not only allows for a better understanding of these microbial interactions but also informs bioprocess optimization design for improved product yields from this specific bioprocess (Ulčar et al.).

Maximizing Product Yield: Increasing the yield of MCCA and diversifying product profiles (for instance by alcohols) are key challenges of chain elongation. By optimizing reactor conditions and substrate utilization, researchers in this Research Topic demonstrated the potential to achieve high-value butanol production from ethanol and acetate and to effectively use endogenously produced H_2_ feedstock. In contrast, other authors used H_2_ and/or CO_2_ as co-substrates and advocated that chain elongation from brewer’s spent grain residues is stimulated by these additional gaseous compounds (Robles et al.; Henry et al.).

CO_2_ as a Versatile Tool: CO_2_ is emerging as a versatile tool for controlling microbial community dynamics and product profiles in ethanol-based chain elongation systems. Through controlled CO_2_ supply, researchers manipulate microbial metabolism to favor specific functional processes like homoacetogenesis, chain elongation and/or solventogenesis. This has allowed CO2 utilization as feedstock, to provide highly selective caproic acid formation, or to promote both butanol and hexanol (de Leeuw et al.).

Taken together, these research endeavors underscore the potential of Microbial Chain Elongation as part of the Carboxylate platform to drive the transition to a sustainable Circular Economy. By harnessing microbial catalysis and ecological principles, we can unlock the valorization of organic waste streams and CO_2_, ushering in new ways for resource efficiency and environmental stewardship. Further elucidation of the mechanisms of microbial communities, which are often used in chain elongation processes is vital. Open research facilities that allow quantitative analysis, deeper understanding and process development, such as those offered by UNLOCK, can be helpful in this regard (Kleerebezem et al., 2021; Wageningen University and Research and Delft University of Technology, 2024). As research in this field continues to evolve, the prospects for scalable and economically viable bioprocesses have become increasingly promising.
